# The Case for Octopus Consciousness: Temporality

**DOI:** 10.3390/neurosci3020018

**Published:** 2022-05-03

**Authors:** Jennifer Mather

**Affiliations:** Department of Psychology, University of Lethbridge, Lethbridge, AB T1K 3M4, Canada; mather@uleth.ca

**Keywords:** consciousness, temporality, octopuses, cephalopods

## Abstract

Temporality is one of the criteria that Birch has advanced for areas of cognitive ability that may underlie animal sentience. An ability to integrate and use information across time must be more than simply learning pieces of information and retrieving them. This paper looks at such wider use of information by octopuses across time. It evaluates accumulation of information about one’s place in space, as used across immediate egocentric localization by cuttlefish and medium distance navigation in octopuses. Information about useful items in the environment can be incorporated for future use by octopuses, including for shelter in antipredator situations. Finding prey is not random but can be predicted by environmental cues, especially by cuttlefish about future contingencies. Finally, the paper examines unlimited associative learning and constraints on learning, and the ability of cephalopods to explore and seek out information, even by play, for future use.

## 1. Introduction

There are many definitions of consciousness and many viewpoints about its functioning. Often paradigms about consciousness are formed for humans, and only later are there attempts to adapt them to non-human animals. Thus one of the formative concepts of human consciousness is the Global Workspace [1]. In this theory, consciousness directs a ‘spotlight’ of attention to particular sensory experiences, which are then processed globally across the brain rather than locally in a particular area. This is the functional equivalent of working memory, when facts and concepts are held for a very short period, integrated and processed before being transmitted into longer-term memory or forgotten. Such a view explicitly makes cognitive ability a foundation for consciousness, although it is sometimes divided into primary consciousness, representing such cognitive activity, and ‘higher-order’ consciousness including metacognition and informing by language. In support of this division, Pepperberg & Lynn [2], among others, suggest that complex cognition can foster sentience but is not the same as consciousness.

Efforts to transform global workspace theory to the foundation for animal consciousness have had only some success. Of course non-human animals cannot report their conscious feelings by language (though see the parrot Alex) and, as Edelman and co-workers point out, we must look for other correlate of this type of consciousness [3]. These authors comment about brain size and areas of control analogous to those of ‘higher’ mammals, not strictly the same areas, though Key [4] tried to use homology rather than analogy to deny fish the ability to feel pain. One possible set of neural correlates of consciousness is [3] re-entrant neural circuits, widespread correlated brain activity and analogous neural structures to reveal sentience in non-mammalian animals, which they found in birds though not necessarily in octopuses. They also evaluated ‘rich discriminatory behavior’ which again they found in birds, and they concluded that evidence for consciousness in cephalopods was simply not present. This led to Mather’s paper [5] pointing out some of the detailed studies that they had missed, and there are [6] parallels in control areas of the octopus brain to those of humans, while recent research has widely expanded our knowledge of cephalopod cognition (e.g., [7]). Yet this evidence has not been fully evaluated, and Birch et al.’s [8] focus on the four areas of behavior—perceptual richness, unity, temporality and affect—offers a way to assess at least primary consciousness free of judgment about subjective experiences.

It could be argued that the basis of sentience is monitoring, broadly conceived, and there are two aspects of such checking/evaluating: monitoring oneself and monitoring externals. Monitoring of the self within the environment [9] becomes important as soon as animals are mobile, and the egocentric view centered on oneself primarily results from receiving (in the case of mammals) input derived from the visual gaze. This egocentric view is transformed in the brain into an allocentric understanding of the three-dimensional environment around oneself, and such information is stored in memory. Moving through the environment, an animal uses its plans for movement to construct a reafferent copy to compare with the updated set of sensory information resulting from the movement [10]. Monitoring external events or items and remembering them are the most obvious integrated uses of this allocentric information about what and where. Two suggestions have been offered for such abilities, with an emphasis on the learning aspect. One is Unlimited Associative Learning, the concept that flexibility in both how and what it learns might indicate that an animal is conscious [11]. By definition, learning is acquiring information and using it across time, but learning varies in its complexity and even very simple animals possess basic types of learning such as habituation. See in [12] the necessity for animals to perform many different types of learning in many different situations. Octopuses fulfil this criterion [13]. It is useful to also think not just about what information any animal acquires but also how it uses it. Another standard for learning achievement, echoing Birch et al.’s [11] criteria, is cognitive complexity. The different cognitive tasks vary in level of complexity and across species [2] and are a reflection of the demands of each one’s habitat. The authors give the example of caching birds, which must remember not only where they stored food but how long it will last and where to find it again by clues from a possibly changing landscape. For a cephalopod comparison, see the demands in a varied environment of predator evasion and food acquisition, particularly in win-switch and selective prey acquisition strategies [14]. One metric is cerebrotypes [15], brain allocation to different areas as a result of ecological niches and habitat complexity, and see [16] for structural detail of brains of octopuses found in different habitats.

Birch et al. [11] suggest two different temporal abilities, representation of past events and preparation for the future, and two field studies will be featured in this paper that provide examples of this kind of complex learning and cognitive actions to evaluate. But there are two more sub-divisions of such learning, ‘where’ in terms of [9] focus on where an animal is within its environment, and ‘what’ in terms of items available for assessment and use. Any mobile animal must remember where it and other items are, thus also integrating information about self and others, and they have many different techniques in what has been called [17] their ‘navigation toolbox’. Yet similarly animals must monitor physical structures in the environment and learn how to adapt their use, including how they can be manipulated, directed by the question [18] “What can I do with this object?” In some ways this evaluation is the mirror image of perceptual richness [19], use of the information that has been gained by the senses.

## 2. Accumulation of Information about Past Events to Guide Movement

Orientation and movement with reference to self in any species can be across several scales: micro in the immediate egocentric orientation to items of interest such as prey, meso within an animal’s normal home range or territory and macro or long distance.

## 3. Short Distance Egocentric Localization

Orientation to items such as prey may be simple, but it involves calculations of sensory information that might not be automatic, especially if the two eyes cooperate to give finer information about item location [20]. For instance, cuttlefish commonly capture prey using ejection of the two very flexible tentacles, similar to capture by the ejection of the sticky tongue of chameleons (e.g., [21]). There are three steps to capture of a shrimp or small fish [22] angular alignment so that the prey is straight ahead, accompanied by convergence of the eyes so that the image of the prey falls on the fovea-like visual streak, distance adjustment so that the prey is a preferred distance away, and the open-loop quick ejection of the tentacles. At first this sequence was thought to be reflex-like. However, the automaticity both of visual stimulus release of the capture attempt and modification of control of timing were changed to add more flexibility by the second month of life, in parallel with the growth of the vertical lobe learning center of the brain [23]. In addition, screening of one eye proved that prey capture was binocularly guided, as it was faster and more accurate when the animal was able to use stereopsis [24]. Finally, these studies had been carried out with stationary prey and when facing moving prey, cuttlefish adopted a variety of aiming tactics [25]. They could swim alongside the prey matching velocities, adapt the striking angle or calculate the future position of the prey and strike at where it would be rather than where it was presently. Are these adjustment cognitively calculated? They may, like human motor routines, be originally carefully directed until learned and automatic, as in car driving.

Egocentric localization may be different in octopuses, which locate prey by chemotactile search but still need to know their location in the environment. They usually employ monocular vision, which is less accurate than binocular in pinpointing location. But monocular animals have a technique to gain more depth/distance information, they can use motion parallax, moving the head so a different view of the landscape is sequentially rather than simultaneously produced [26]. Octopuses gain motion parallax by a vertical Head Bob when faced with an object or situation ’of interest’. This as an example [5] of them using a Spotlight of Attention, ref. [1] as the octopus’ reaction to an unusual situation was not to respond, but to focus on getting more information about it, definitely guided by primary consciousness. Given what the cuttlefish can calculate about position, it seems likely that the octopus can also gather a fair amount of position information using these techniques.

## 4. Medium Distance ‘Mapping’ of Self in Environment

Any animal moving in its home range must gather a wide variety of information about its own position and that of items of interest in its environment [17] using its ‘navigational toolbox’, and remember it for future actions. This is true for octopuses as well as honeybee foragers, caching birds, and monkeys moving out from nighttime sleeping places, but it takes careful detailed observation to record it.

In the mid-1980s, a volunteer team did an observational research study of octopuses’ behavior in the shallows (intertidal to 2 m depth) off the shore in Bermuda [27,28]. With many individual observers, the team was able to follow two octopuses all the daytime, from 0630 to 1830. While animals were mostly not identified individually, octopuses stayed in one sheltering ‘den’ for an average of 10 days [29], and the observers were especially able to follow one individual for seven consecutive days. It quickly became apparent that the octopuses were Central Place Foragers [30], making short or long trips across the rock, rubble and sand surround and returning to their shelter after a foraging duration of minutes to hours [28]. Many of the trips moved the octopus well out of sight of the den (see Figure 1 in the paper cited), and octopuses did not use chemical cues from the substrate to retrace their outward path but jetted through the water. Their integrated use of information about their environment became apparent as subsequent returns to the den were traced [28]. They possessed spatial memory and were remembering their previous searches across the sea bottom. One ‘acid test’ of how an animal remembers its place in the habitat is displacement, when it is moved to somewhere it had not planned to go [31]. During the several years of the study, 11 instances of displacement from their path were noted. In only one instance did the octopus find and retrace its outward path to the shelter. Otherwise they went directly home from a different heading than they would have originally used. This understanding of their location strongly suggested that they had constructed some kind of cognitive map [32] of their 200 m square home range.

A subsequent laboratory study, though only of a few individuals, showed that they could learn to orient to visual targets as a beacon, to use a larger primary one to orient to a smaller secondary item, and to follow the beacon when it was moved to a new location. Interestingly, while octopuses foraged in the areas relatively close to the den, they were selective in the microhabitat they chose. They used vision to move to ‘likely locations’ such as cliff edges, crevices and large rocks [27], then foraged by intense chemo-tactile exploration. Leite et al. [33], doing much the same work but without the large team, saw some variation in search behavior sequences depending on microhabitat, with octopuses using a saltatory stop-and-go search strategy [34] but also taking advantage of unusual circumstances such as a fish sheltering in a crevice. Having acquired their mostly small crustacean and molluscan prey, octopuses either chose to shelter in a local crevice to consume it or took it back to the den, and the significant predictor of this choice was the distance to the den [27]. The animal that was followed for a week left the den in one direction for most of its searches in one day and then chose a different direction in subsequent days (see Figure 1 in the paper cited). This choice indicated a win-switch foraging strategy [35]. These observations also suggested episodic-like memory [36], an ability which was not thought possible for animals, especially invertebrates, at the time and which is still sometimes challenged (but see [37]).

Laboratory studies gradually confirmed what had been seen in the field. Boal et al. [38] used a scenario that somewhat replicated the situation in the normal environment. They placed octopuses in an open circular arena with a shelter of a few bricks and two recessed jars, with one upside down and blocked and one open for escape from bright light and a shallow water level. They confirmed that the octopuses sheltered in the bricks but then explored the arena by moving out from this home by forays in different directions for 72 h, with half of the exploration taking place in the first day. Octopuses quickly learned to move to the open burrow, shortening the duration of movement over three trials, and retained this memory for at least a week. When the former open burrow was blocked but a new one was opened, octopuses’ time to find shelter increased, but it returned to its former duration within another three trials. This is a good experimental situation as it avoids problems with food rewards and satiation, and it clearly demonstrated that octopuses knew their location and that of important items, as in the field work [28]. A continuation of the test series with octopuses [39] gave them visual cues to indicate the presence of the open or blocked burrow. The arrangement of the visual cue items around the opening was different when one burrow was open than when the other was open. Octopuses were able to learn to find the correct burrow given the specific set of visual cues. In other words, they had performed conditional discrimination. This could parallel a situation in the intertidal environment, where one set of cues would be visible when the tide was in and another when the tide was out.

Meanwhile, Karson et al. [40] were setting up a similar experimental situation for cuttlefish. A location in the bottom of a tank was not favourable for escape with these species, but an opening in a vertical wall proved to be a good parallel. These openings could be surrounded by fabric of different patterns to provide a visual cue for their location. Cuttlefish learned to swim through the door indicated by a visual pattern, often performing well after one set of three trials. It was not difficult to switch the fabric location to produce reversals, and the cuttles learned the correct choice through several reversals. By the sixth reversal, two of the animals had near-perfect performance after the switch, some of them being able to choose correctly when the cue location switched, clearly guided by cue location. When they used cuttlefish in a parallel to the octopuses with both items and fabric as visual cues to the location of the correct opening in the maze wall, animals could also learn this, becoming faster and more consistent in their exit times. Again the animals learn a reversal where the same cues indicated a different opening randomly from trial to trial. This is a practical necessity in the field, as on returning from a foraging trip by a different direction [27], an animal would need to understand an apparently different cue configuration. Next the authors used a modified testing arena so that cuttlefish were required to turn left or right in a T maze to gain access to a dark sandy location, which they preferred [40]. The turn direction (against their initial preference) and the position of salient visual cues were the same. After training, the visual cues were moved to test whether turn direction or cue location were more important to them. Five cuttlefish followed the cues and two continued the same turn direction, indicating they were guided by vision, but not solely.

Teaming up with Nikki Clayton, who had previously tested [41] for episodic-like memory in caching birds, Jozet-Alves [37] evaluated cuttlefish for episodic-like memory using a more difficult task. Episodic-like memory is tested by a combination of stimulus type (what—prey type), where (the location of visual cues) and when (lag of one or three hours after previous prey was supplied). Cuttlefish were tested for prey preference (crab vs. shrimp), first rewarded by food at the location of one of two visual cues that nevertheless were moved around their tank, learning where to go to get which reward. They were given the non-preferred prey beside its visual cue one hour after a previous feeding, but then given the preferred type, again at its correct cue, only three hours afterwards, and they learned where to go to take which reward, depending on the time lag. This is a very sophisticated memory ability, though it might be argued that it is a type that win-switch foragers need to learn, that prey is not immediately replaced in the same location. The cuttlefish’s ability to sort out all this different information about cues, spatial location and timing of prey availability suggests at least simple consciousness. Although there is much more to learn about this ability, there has been no follow-up from the demonstration of episodic memory [37], although studies of rat spatial orientation [36] show what might be investigated. One suggested avenue for investigation of their spatial memory follows Cartron et al.’s [42] finding that cuttlefish can navigate in a Y maze using both landmarks and the plane of polarization of light. When given a choice they may rely more on the e-vector of the light than on landmark location. Since all the studies reported here looked at landmarks alone, much more work remains to be done to sort out responses to both types of visual cue.

## 5. Long Distance Migration

Some cephalopods such as squid [43] and cuttlefish [44] move to and gather in large numbers in restricted areas to mate and deposit eggs or egg clusters. Shallow-water octopuses may move offshore and return to the shallows to mate and lay eggs. These migrations at the end of the lifespan may go to locations where the animals have not previously been (as in bird migration). They are likely not learned, rather dependent on some inner drive, so sentience is thus likely not involved. However, these long distance movements have been extremely poorly investigated.

## 6. Accumulation of Information about Items in the Environment for Future Use

### 6.1. Avoiding Predation

The soft-bodied octopus is particularly vulnerable to predators and avoids being captured by a series of escalating anti-predator strategies [14], from sheltering out of view to camouflaging itself, to startle, and finally to escape and ejecting repelling and concealing ink. In 2009 the scientific world was startled by a report of a series of incidents in which octopuses in the field gathered the split halves of coconut shells, cleaned out the sand, and took them under their arm web as they moved out over the soft substrate, presumably to hunt for prey [45]. After a trip of some time and distance, an octopus would stop, pull the coconut halves out from under the arm web, reassemble them, and crawl inside to use the shells as a shelter. While the paper had no quantitative results and no follow-up assessments of circumstances have been done, this was undoubtedly predictive behavior, gathering the coconut halves and transporting them for a future use.

Why would octopuses show such behavior and what does it tell us about utilization of items in the environment? Octopuses must be able to imagine the transformation of buried coconut halves to cleaned-out joined ones and also predict that these will fulfill a need for shelter in an area about which they must have some previous knowledge and to which they plan to go to in the future. Although *Octopus kaurna* can burrow in the substrate [46], most octopus species cannot and are dependent on hard substrate or solid shelter to take this first protective step and protect themselves when resting. Their distribution is considered to be limited to these areas or by the presence of such structures [29,47,48,49,50,51]. When solid shelters are not available because of substrate type, octopuses may use human-provided shelter such as beer bottles [52], tires [53] and plastic waste [54]. An extreme lack of appropriate solid shelter in areas of sand and mud may lead to grouping in the only available locations such as ‘Octopolis’, at which point octopuses express ‘semi-social’ behavior [55]. In a similar substrate, octopuses given an excess of provisioned shelter choose not to stay near one another [56], so they were not clustered due to sociality. They are thus not attracted to one another but to places to hide. Not being able to find optimal shelter can have consequences, as female octopuses with less space may lay fewer eggs and thus have decreased fecundity. So this ability to carry shelter with them can reasonably be seen to be selected for, but is this ability to predict future consequences more general?

Safe in our human enclaves and having killed off or excluded most of our predators, we forget how pervasive the threat of predation is to nearly all animals. With a lifetime reproduction rate ranging from around 100 for big-egged octopuses to tens of thousands for small-egged ones, and one or two hundred for squid and cuttlefish, attrition over the lifespan that would result in four or five reproducing adults must be very high—though attrition of the tiny paralarvae is probably the main culling factor in small-egged octopuses. Survival is so important that octopuses have an array of strategies, ranging from long-term behavioral patterns to predator encounter responses (see Table 1 for some common ones), with mechanisms that likely range from pre-programmed automatic responses to learned ones. Does this mean that predators always kill an octopus that they attack, who thus have no opportunity to learn about potential risks? By no means, octopuses in particular often escape with damage to or loss of one or more arms, which can regrow, as 60% of the octopuses of several species whose bodies were examined from collections had damage to at least one arm [57]. In fact, autotomy of one or more arms is an ultimate survival strategy, and long-armed species such as *Abdopus* have areas of weakness where arms are released in times of predator attack [58]. The squid *Octopoteuthis* releases arms at predator attack, and these wriggle and emit light. This would be a distraction and also give the predator an assured but smaller meal white the squid escaped, leading to its survival [59]. However, the variety of antipredator actions and the time scale over which they are prepared means that it is difficult to know not only whether they were learned but also whether the animals were conscious of the strategy choices. Laboratory studies confine the animals and make escape less likely so that results may be invalid. Animal welfare regulations generally prevent researchers from proving failure of evasive strategies and dooming their animals to death (though see [60]). Actual predation is seldom seen in the wild, and field studies are selective and lack any controls, and the presence of observers may offer animal subjects some protection. So more than any other area discussed here, future prediction for predator evasion is difficult to evaluate.

The longest-term actions that octopuses can take is to not be available for capture, and octopuses evade predation by not being out of protective shelter much of the time. They are often described as time-minimizing foragers, minimizing risk [33]. Field observations showed that *O. insularis* made many short foraging trips, capturing relatively small prey while showing excellent habitat-matching camouflage, and returning either to their permanent shelter or protective niches to consume it. And this is why octopuses would carry coconut shells out into the open with them, for shelter [45]. Still, giant Pacific octopuses (GPO) instead may be rate-maximizing foragers emphasizing maximizing energy intake. They narrow their prey choice in productive areas and maximize prey size where possible [55], but adult GPOs weight 25 kg at adulthood and have far fewer potential predators than the small species.

Only one study evaluated whether octopuses changed activity time and amount in response to predator pressure. Meisel et al. [61] kept octopuses for several days in an aquarium with a plexiglass divider between them and either a moray eel or a triggerfish, and monitored their activity cycles. Octopuses were out less often and shifted more to nocturnal activity in the presence of the diurnal open-water triggerfish. But they did not do so in the presence of the nocturnal narrow-bodied moray, which is known to snake through the rocky substrate and capture octopuses in hiding [70] so time of its activity was less relevant to the octopus. Clearly this set of adjustments was learned, but whether it was also conscious is difficult to know.

When actually encountering a predator, prey species tend to escalate anti-predator strategies, and cephalopods are only special in that they tend to have several, moving from appearance matching to appearance change, to attempts to startle and to flight, sometimes accompanied by ink ejection [14]. Such a sequence was only traced by Langridge [71] for cuttlefish and will be discussed after the steps themselves have been described. Appearance is the first aspect of antipredator behavior in cephalopods, and it precedes any predator approach. When cephs are out of shelter, they manipulate their appearance so as not to be noticed. This background matching has been particularly well studied in the skin responses to sand/rubble grain size in cuttlefish [72] where different skin patterns seem to be almost automatically assumed on backgrounds of sand, pebbles or rocks. This homogeneous response is likely only true for homogeneous backgrounds, as octopuses on a varied rocky background heterogeneously matched predominant features [62]. Cuttlefish matching also includes raising the papillae of the skin to match background irregularities [73]. Not having a background on which to shelter, open ocean squid generate countershading, darkening above and paling below to match the luminance level of the down-dwelling light in midwater. However, this adjustment is reflex, controlled by feedback from opsins in the skin [74]. The source of control for the other adjustments is not known, although the animals do not appear to get visual feedback to monitor the patterns they produce.

When out of shelter, cephalopods may resemble specific features of the landscape. Cuttlefish can place their arms in a linear position angling away from the body [75] that matches the angle of sea grasses behind them. Octopuses [65,76,77] can locomote with the fourth arm pair and place the other in positions and with displays that resemble algae. *Abdopus* in particular hunches down and tiptoes slowly on the fourth pair of arms, swaying as if moved by water currents, an action nicknamed ‘moving rock’. Both *Thaumoctopus*, referred to as the mimic octopus, and *Abdopus* seem to copy other species, particularly those that might be dangerous or repellent. There was, however, a spate of papers stating “I though it looked like xxxx” without any proof that the suggested models even lived in the same habitat. Norman [66] noted that mimic octopuses resembled in turn a lionfish, a flounder and a sea snake, and Huffard et al. [78] traced the phylogenetic extent of the flounder-mimicking octopuses. They found there was a group of related species that all had long arms that might have ‘flowed’ behind them when they were moving head-first across the sand and monitoring for prey as they went, an adaptation that could have preceded camouflaging action. Hanlon et al. [79] looked deeper into the resemblance. All in all, the answer is that there is some mimicking capacity, but that it is an automatic response to threat, not a targeted response to the sight of a particular model to mimic, and thus not primed by memory and awareness. A different view emerged when researchers watched octopuses forage across the landscape and then chased them. The animals made many different patterns, not necessarily camouflaging ones, which would confuse a visual predator who was looking for a particular appearance. Predators often form a Search Image in memory and use it to locate prey that match a remembered static color morph [80]. However, cuttlefish simply moving across the landscape did not have conspicuous patterns [81] so the context for the two studies may have been different. But what does this list of possible mimicry tell us? Not much, because it’s difficult to tell how the appearances were programmed, and laboratory studies are necessary. Looking at the series of antipredator responses tells us more.

Animals that are vulnerable to predators do two things: they sort out the potential threat of different predators and they respond differently to the amount of threat that is posed by a particular approaching animal. Thus different cephalopods can produce the sequence: from hiding to camouflage to action. But is it automatic, or planned and thus integrated across time? The only study to investigate this question was Langridge’s [82] for cuttlefish and the answer was clear: it is pre-planned. To ‘serious’ predators, escape and hide form a set of responses in squid [60,83]. They responded to different predatory types of fish by being in different areas where they would not be located and fleeing at their approach, although this study confined them in a laboratory tank and this may have biased the results. Cuttlefish respond to ‘medium threat’ predators with a sequence of displays before resorting to flight [71], and the sequence was different for different predators. In particular, the deimatic eye-spot display was not part of the sequence when cuttlefish were approached by dogfish and crabs, who do not rely on visual cues for predation. In other words, the cuttlefish had learned to omit the display because they knew these species do not see and respond to it. The sequence is not pre-programmed but based on what responses the animals have learned is effective. Caribbean reef squid also carefully calculate displays to approaching (again not seriously dangerous) fish [84], including an agonistic Zebra display generally addressed to conspecifics [85]. They may ignore, evade or generate eye spot displays to the fish around them, and the cues (size, distance, speed of approach) given out by different species were responded to differently. Squid have four different eye-spot locations to ‘call on’ for the deimatic display and they chose two of the four, partly dependent on the direction of approach of the fish. Both the cuttlefish and the squid were carefully calculating risk before choosing responses, and octopuses likely have a similar sequence.

What about the responses to really serious threats? Escape is the obvious answer and Caribbean reef squid are very ‘jumpy’ out in the open, they jet away an average of eight times an hour, though they only go a few metres [84]. Jetting away obviously expends energy and disrupts the life of animals, but these squid feed at night and only rest in the daytime except for adult courtship. Although cuttlefish camouflage is complex and well understood, this species normally prefers to hide by digging into sand in the daytime, if given a choice of substrates [86]. Even though the skin patterning may not normally be used, it is a very sophisticated match. What about inking? *Lolliguncula* squid were much more likely to use ink as predators approached more closely, and paralarvae used stereotyped responses and juveniles and adults used planned ones [87].

Ink is an effective deterrent, used by *O. bocki* against hatchling turtles [68]. Ink is very effective because the melanin in it is both irritating and confusing to predators and a warning signal to conspecifics in squid [88,89,90]. If ink is exhausted, it takes 30 days to replenish, and fully discharging ink may cause damage to the squid itself [91]. Thus squid ink may be used sparingly, lest there is none when it is needed in the future, but we have no idea whether this is monitored and planned. There is also the curious case of the ink ‘pseudomorph’. Ink can be used for screening from predators, but it can also be bound together by mucus, and squid can use this as a ‘decoy’ in a complex sequence of actions described in *Idiosepius* [92]. The squid ejected a pseudomroph of ink, then jetted away, sometimes using a sequence of several ejections, sometimes changing direction or stopping and turning pale after ink ejection, leaving the sculpin fish predator to attack the ink and not the squid. 

The authors of this study commented that whether the sequence was fixed or variable had not been determined, but Anderson and I found a clear confirmation of variability in similar behaviors for *Euprymna* squid [93]. When we were attempting to capture these small (<2 cm mantle length) and non-streamlined sepiolids, we were struck by the variety and unpredictability of their escape responses, which seemed a larger repertoire in adults than juveniles. Squid could produce the sequence described above but they also could turn dark, place their arms in camouflaging postures and move to the water surface imitating algae, become transparent with elongate arms and float mid-water disappearing from view, or sink to the bottom and either camouflage against pebbles or bury in the sand. Their displacement was saltatory, either moving quickly or stationary, which helps to evade predator notice, as is also seen in *Idiosepius* and in cuttlefish [94]. The squid appeared to note the failure of a particular evasion tactic and so tried another, and the same range of postures, patterns and actions was widely preserved across the coleoids. Planning, both short and long term, was necessary for survival.

### 6.2. Finding and Manipulating Prey

The second situation in which to look for learning and assessing consequences in the future is one vital to any species, finding food. Ecological researchers seem to take it for granted that food choice by non-human animals will be based on the simple metric of energy gained, resulting from energy produced by digestion—energy expended in finding, preparing and eating. However, this is not always the case for octopuses, as is seen in a very careful energetic study of *O. rubescens* consuming two prey species, a crab and a clam [95]. The authors measured the octopus’ handling time and its energetic expenditure during this interval, carefully calculated the prey mass and its energy value.

The crabs were preferred by a ratio of 3:1, but the energy expended in eating them was double that of the clam and the energy gained was less. Crabs dominate the diet of many octopus species [28,96,97,98,99], and early exposure to alternate prey cannot reverse a crab preference in *O. maya* [100], so these choices seem to be ingrained. Thus many different types of influence, not just energetics, shape prey preferences.

Carefully controlled laboratory studies show nuances of learning that shaped octopus and cuttlefish prey choices in both short and longer-term learning. Schnell et al. showed that cuttlefish can learn to delay gratification, waiting from 50 to 130 s to receive a preferred prey, in contrast to a less preferred one available immediately, in a choice paradigm [101]. Cuttlefish preferred two shrimp rather than one, but after a sequence of one vs. zero, they were ‘used to’ the single and took it instead of the two [102], an unrewarding preference that receded over an hour’s duration. When cuttlefish were cued by different visual backgrounds to predict prey presentation by a visual or olfactory cue, all of them could remember the correct sensory predictor for an hour and many until the next day [103]. They could also remember that a reward would be coming in the future [104], consuming crabs in the daytime when shrimp would not be delivered the next evening but leaving the crabs when the shrimp was due to be produced later. With an even more subtle manipulation, the cuttlefish preferred predictability, and would leave the crab when a shrimp was reliably delivered the next evening yet consume it when delivery of this preferred prey was random. Some central organizer was calculating and using information about availability and preferences from the past to generate future choices.

Studies that were obviously not carefully controlled nevertheless showed the influence of learning on octopus field choice of prey species, as selection of prey type was responsive to more than general prey availability. Ambrose [96] conducted preference tests in the laboratory and observed actual choices in the field. His octopuses preferred crustaceans and avoided one *Tegula* snail species. But in the ocean snails were abundant and crabs scarce, so the octopuses ‘settled for’ predominantly another more tolerated *Tegula* species. Experience with surface texture could reverse a preference, as giant Pacific octopuses preferred crustaceans yet avoided a ‘hairy’ species [97]. Collection of remains of the prey of many members of one species led researchers to conclude that octopuses were generalist predators, yet examination of prey remains from individual octopuses turned up many specialists [105], likely due to learning. Sequences of shells collected daily were sometimes from a single prey species, perhaps one living in a specific habitat such as a small sandy area (also suggested by Hartwick et al. [47] based on indirect evidence). This led the authors to conclude that an octopus had found and returned to a ‘good location’ for preferred prey, also integrating information about its spatial location. Octopus personalities, presumably affecting exploration and thus range of opportunities to find new prey, could also affect the variety of prey species taken and remembered [106]. This generalist-specialist mix occurred both across octopus species [107] and within species in different habits [108]. In a rich background of mixed habitat and many prey species, different individual octopuses flexibly used many sources of information.

Even when an octopus dug out molluscan prey from the sand or found it under a rock or in a crevice, its soft body was not yet available for the octopuses to consume, and they had to learn handling techniques. In the field, Ambrose & Nelson [109] noticed a wide array of molluscan species at octopus homes had neat small holes in the shells, and it was obvious that they were located at different areas on the shells of different species. Nixon [110] spent years investigating the drilling process and the holes, finding that a combination of rasping by the radula (a ribbon of teeth) and the salivary papilla produced the hole. After that, injecting venom from the posterior salivary gland would weaken or paralyze prey muscles. In a pioneering though simple lab evaluation of the drilling process, Wodinsky [111] saw that octopuses would first try to pull snail prey out of its gastropod shell but when that failed, they would resort to drilling. The hole locations were not random, because when acrylic was used to cover the spire of the shell, the octopus drilled as close to its edge as possible [112]. This was a two-step process, pulling first and drilling if the pull did not succeed [113]. Both a minimal thickness of the shell at sutures and the location of retractor or adductor muscles underneath were favorable cues for choice of drilling location [114]. Muscle localization for drilling might need to be fairly specific, given that 2/3 of the area within bivalve shells is enclosed water, and was aimed close to the atypically located retractor muscle of cowries [115]. This had to be learned from the outside and by trial and error. *Venerupis* clam valves were generally pulled apart, but when they had been wired shut, the octopuses drilled them or chipped a little of the valve edge off, and in both cases injected poison [116]. Given mussels, *Venerupis* and the thick *Protothaca* clam, octopuses chose the first two species. But when all clams were opened and delivered ‘on the half shell’, the thin shelled mussels were nearly totally ignored. Drilling the heavy shell was avoided but apparently, as opposite from the preference for crabs, the mussels didn’t ‘taste good’. Were drilling locations learned? Merlino [117] was able to find juvenile octopuses that had never seen a mollusc as prey. He confirmed that the first few holes drilled were near the snail aperture and nowhere ‘practical’ for prey paralysis, but that within a few attempts the octopuses had learned the proper locations on the shell. It seemed to be a rapid and somewhat guided learning.

### 6.3. Guidance of Learning

Although Ginsberg & Jablonka [12] presented the case for unlimited associative learning as a foundation for consciousness all species, including humans, have some limitations on learning. Relying on learning is evolutionarily expensive as it might fatally fail, and dependence on reflexes may be useful especially at the beginning of the lifespan. Such changes in learning capacity across an animal’s lifespan are of great interest to neurobiologists as demonstrating brain-behavior interactions. How can the cephalopods survive if they must learn what is suitable to catch and eat as well as how to avoid being caught and eaten themselves? To some extent, the answer is by selection; octopuses produce between a hundred or so and tens of thousands of eggs, and very few need survive to adulthood. to continue the species. There are two models of guided learning-reflex mixtures. One program model is a switch over from stereotyped responses when very young followed by reprogramming and gradual acquisition of the flexibility to become a learning specialist and choice generalist at adulthood. The second program model is imprinting, quick and focused learning of responses by stimuli received even before birth/hatching (as in chicks hearing their mothers’ calls in the egg). Most of the work in cephalopods in this area has been done with common cuttlefish who, besides being readily available, have relatively large eggs of around 1 cm in length.

Cuttlefish demonstrate both types of guidance [22]. Cuttlefish were tested with what has been called the prawn-in-a-tube situation. The visual stimulus of a shrimp ‘releases’ a tentacle strike. Animals needed to learn not to strike with their tentacles at a shrimp which had been enclosed in a glass test tube and therefore could not be captured. Adults learned to not strike, although there was a dual retention period of up to 20 min (short term memory) and 60 min plus (long term memory). However, cuttlefish 8 days old had the short but not the long-term memory capacity [118]. Further testing showed that the long-term capacity gradually emerged when animals were 60 to 90 days of age. During this time, the vertical and superior frontal lobes of the brain grew and developed to adult size, so these areas were needed for active storage of information i.e., learning. Since ablation of the vertical lobe area slows or stops learning in adult octopuses [119,120], there is an obvious parallel in the learning function of the vertical lobe in both cephalopod groups.

While newly hatched cuttlefish can survive with this limited memory, how can they be cued to the specific stimuli in their immediate environment? The answer appears to be in the second model, by imprinting, early non-reversible learning in a short ‘sensitive’ period. Imprinting in mammals is generally focused on fixing one’s species identity for adult reproductive behavior, but in some reptiles [121] and in mostly solitary cephalopods, it is more tuned to critical survival function such as predator evaluation and food choice. Early exposure of cuttlefish to crabs, right after hatching, can change prey preference from the normal shrimp to crabs, even though the hatchlings do not start feeding for several more days [122]. When exposed to sand for burying, hatchlings only gradually dig in over the first week [123], but exposed hatchlings are quicker to bury in a novel situation.

Cuttlefish eggs are originally colored with ink from the mother, but gradually become transparent, so the embryos can see out and researchers can observe embryonic mantle contraction rates. Well before hatching, embryos respond with a change in respiration rate to cues of danger, such as a prick on the egg or predator odor; later also to sudden bright light [124]. When exposed to the sight of non-preferred crab prey in the egg, cuttlefish change their choice of prey from shrimp to crab [125]. If exposed to predator odor as embryos, they delay hatching and use stronger disruptive body patterns and dig in sand sooner after they do hatch [126,127]. Therefore this sensitive period starts before hatching. Hatching is recognized as one of the critical transitions in cephalopod development [128], exposing the new hatchlings to the imminent risk of predation and in many cases thrusting them into open water for the pelagic paralarval stage. But clearly for big-egged squid and some octopus species, the transition is not as abrupt as it seems, and some learned preparation smooths the way. The capacity to learn is not the same across the lifespan, although we neither understand the neural changes that must accompany it nor do we know how life history might affect the consciousness we hope to understand.

### 6.4. Exploration and Play

The lives of both human and non-human animals do not consist only of immediate decisions shaped by a narrow set of stimuli, but instead involve what Wilson et al. [129] discuss as an exploration-exploitation trade-off. Sometimes the animal needs to gain information about its environment and sometimes it focuses on using it. Many animals show this trade-off, but in different schedules. For instance, forager bees first make a set of flights from their hive, some short range and some longer and in random directions [130], in order to learn about the environment. They then switch to longer, well-directed flights to gather food. Rats choose a home base and make forays from it [131], with tracks extending over time and moving faster when they pass over known areas. Casual observation shows that an initial period of exploration is true for octopuses put into a new captive environment, and this burst of exploratory activity has been recorded in cuttlefish [40]. One of the strategies suggested for reaching this exploration-exploitation balance was ‘directed exploration’ [129]. Octopuses, and most generalist foragers, first go to microhabitats likely to contain prey and then explore within them [14], finding particular prey in specific substrate types and returning later for more—or switching after failure. They evidently have the cognitive ability and perhaps consciousness to calculate this balance.

Gaining data from the environment can move beyond static information gathering to play, evaluating not just ‘what can this object do’ but also ‘what can I do with this object’ [18]. Both exploration and play occur irregularly, have complex sets of behavior and many possible stimuli and are easily interrupted. Play produces many incomplete action sequences and its survival value is not immediately apparent [132]; it is suggested as an indication of consciousness. Play in birds is seen primarily in corvids and parrots, but corvids play during development and parrots continue into adulthood [133]. The authors concluded that the birds that continued to play were generalist foragers that lived within a complex environment and needed to learn new opportunities and use new skills. These conditions are interesting because adult octopuses perform object play according to Burghardt’s [132] criteria. This was first discovered when testing them with a floating pill bottle in a barren aquarium with a slow water flow [134]. A couple of the octopuses repeatedly blew jets of water to move the bottle to the intake location, where the water flow returned it to the animal, the aquatic equivalent of bouncing a ball. Kuba et al. [135] extended this line of investigation, giving octopuses a plastic toy object and watching its manipulation by the octopuses’ arms. The authors set a sequence of involvement, from 0 = no movement to 4 or 5 repetitions of the same action unit, and the units were Tow, Pass from arm to arm, and Push-pull the item with one arm. They first confirmed that octopuses discriminated between food and non-food items: food was passed to the mouth (level 2) and eaten, then its containers were subsequently ignored, whereas non-food was manipulated for short bursts of time and more frequently. For both octopus species there was a sequence of actions over repeated trials, as they initially explored, then habituated to the item and later returned to play-like interactions. Like parrots [133], octopuses continued to play past the juvenile period. Pisula [136] concludes that animals who play must have both big brains and complex control systems, which is true for octopuses, and it is particularly noticeable that they could perform playful behavior both by water jet expulsion and arm movement. Does that mean octopuses must have consciousness to guide this complex set of interactions?

## 7. Conclusions

While this account follows Birch et al.’s [8] third area, temporality, for investigation of animal consciousness, it reveals that octopuses do far more than acquire information at one time to be used later. They gather information about their environment and may make a kind of cognitive map to guide movement, foresee challenges and arrange protection, and balance many influences in acquiring prey. Octopuses and other cephalopods flexibly use a suite of antipredator behaviors and balance predator avoidance with acquisition of prey. The kind of monitoring that is revealed here transcends model like that of Baars’ [1] spotlight of attention, it is not just focusing attention briefly at one time. Peters [137] suggest humans and other animals use attention (focusing on what information needs to be acquired), learning (acquiring it) and habituation (ignoring information that does not need to be acquired) to manage information across time. Octopuses use many different types and sources of learned information, yet such learning is, as in all species, somewhat guided. Finally, the capacity not just to acquire information and store it across time but to guide information extraction when the octopus is producing motor play goes beyond a cognitive foundation, perhaps to consciousness.

## Figures and Tables

**Table 1:** Octopus antipredator responses.

Strategy	Species	References
Hiding	Many octopus species	Ambrose [48], Hartwick et al. [47]
Change activity	*Octopus vulgaris*	Meisel et al. [61]
Appearance		
camouflage	*Octopus vulgaris*	Josef et al. [62]
change	*Octopus cyanea*	Hanlon et al. [63]
passing cloud	*Octopus cyanea*	Mather & Mather [64]
dymantic	*Octopus vulgaris*	Packard & Sanders [65]
mimicry	*Thaumoctopus mimicus*	Norman [66]
warning	*Hapalochlaena maculosa*	Mathger [67]
Arm autotomy	*Abdopus aculeatus*	Alupay [58]
Ink ejection	*Octopus bocki*	Caldwell [68]
Jet escape	*Amphioctopus marginatus*	Sreeja & Bijukumar [69]

## Data Availability

Not applicable.

## References

[1] Baars B.J. (1997). In the theatre of consciousness. Global workspace theory, a rigorous scientific theory of consciousness. J. Conscious. Stud..

[2] Pepperberg I.M., Lynn S.K. (2000). Possible levels of animal consciousness with reference to grey parrots (*Psittacus erithacus*). Am. Zool..

[3] Edelman D.B., Baars B.J., Seth A.K. (2005). Identifying hallmarks of consciousness in non-mammalian species. Conscious. Cogn..

[4] Key B. (2016). Why fish do not feel pain. Anim. Sentience.

[5] Mather J.A. (2008). Cephalopod consciousness: Behavioral evidence. Conscious. Cogn..

[6] Shigeno S., Andrews P.L., Ponte G., Fiorito G. (2018). Cephalopod brains: An overview of current knowledge to facilitate comparison with vertebrates. Front. Physiol..

[7] O’Brien C.E., Ponte G., Fiorito G., Choe J.C. (2019). Octopus. Encyclopedia of Animal Behavior.

[8] Birch J., Schnell A.K., Clayton N.S. (2020). Dimensions of animal consciousness. Trends Cogn. Sci..

[9] Land M.F. (2012). The operation of the visual system in relation to action. Curr. Biol..

[10] von Holst E., Mittelstaedt H. (1950). Das reafferenzprinzip. Naturwissenschaften.

[11] Birch J., Ginsburg S., Jablonka E. (2020). Unlimited associative learning and the origins of consciousness: A primer and some predictions. Biol. Philos..

[12] Ginsburg S., Jablonka E. (2019). The Evolution of the Sensitive Soul: Learning and the Origins of Consciousness.

[13] Mather J.A., Dickel L. (2017). Cephalopod complex cognition. Curr. Opin. Behav. Sci..

[14] Mather J.A., Leite T.S., Anderson R.C., Wood J.B., Darmaillacq A.-S., Dickel L., Mather J. (2014). Foraging and cognitive competence in octopuses. Cephalopod Cognition.

[15] Ponte G., Taite M., Borrelli L., Tarallo A., Allcock A.L., Fiorito G. (2020). Cerebrotypes in cephalopods: Brain diversity and its correlation with species habits, life history, and physiological adaptations. Front. Neuroanat..

[16] Chung W.-S., Kurniawan N.D., Marshall N.J. (2021). Comparative brain structure and visual processing in octopus from different habitats. Curr. Biol..

[17] Wiener J., Shettleworth S., Bingman V.P., Cheng K., Healy S., Jacobs L.F., Jeffery K.J., Mallot H.A., Menzel R., Newcombe N.S. (2011). A synthesis. Animal Thinking: Contemporary Issues in Comparative Cognition.

[18] Hutt C., Bhavnani R. (1972). Predictions from play. Nature.

[19] Mather J. (2021). Octopus consciousness: The role of perceptual richness. NeuroSci.

[20] Read J.C. (2021). Binocular vision and stereopsis across the animal kingdom. Annu. Rev. Vis. Sci..

[21] Herrel A., Deban S.M., Schaerlaeken V., Timmermans J.-P., Adriaens D. (2009). Are morphological specializations of the hyolingual system in chameleons and salamanders tuned to demands on performance?. Physiol. Biochem. Zool..

[22] Messenger J.B. (1968). The visual attack of the cuttlefish, *Sepia officinalis*. Anim. Behav..

[23] Dickel L., Chichery M.-P., Chichery R. (1997). Postembryonic maturation of the vertical lobe complex and early development of predatory behavior in the cuttlefish (*Sepia officinalis*). Neurobiol. Learn. Mem..

[24] Feord R., Sumner M., Pusdekar S., Kalra L., Gonzalez-Bellido P., Wardill T.J. (2020). Cuttlefish use stereopsis to strike at prey. Sci. Adv..

[25] Wu J.J.-S., Hung A., Lin Y.-C., Chiao C.-C. (2020). Visual attack on the moving prey by cuttlefish. Front. Physiol..

[26] Kral K. (2003). Behavioural–analytical studies of the role of head movements in depth perception in insects, birds and mammals. Behav. Process..

[27] Mather J.A. (1991). Foraging, feeding and prey remains in middens of juvenile *Octopus vulgaris* (Mollusca: Cephalopoda). J. Zool..

[28] Mather J.A. (1991). Navigation by spatial memory and use of visual landmarks in octopuses. J. Comp. Physiol. A.

[29] Mather J.A. (1994). ‘Home’ choice and modification by juvenile *Octopus vulgaris* (Mollusca: Cephalopoda): Specialized intelligence and tool use?. J. Zool..

[30] Olsson O., Brown J.S., Helf K.L. (2008). A guide to central place effects in foraging. Theor. Popul. Biol..

[31] Collett T.S., Graham P. (2004). Animal navigation: Path integration, visual landmarks and cognitive maps. Curr. Biol..

[32] Miller N. (2021). Taking shortcuts in the study of cognitive maps. Learn. Behav..

[33] Leite T.S., Haimovici M., Mather J.A. (2009). *Octopus insularis* (Octopodidae), evidences of a specialized predator and a time-minimizing hunter. Mar. Biol..

[34] O’Brien W.J., Browman H.I., Evans B.I. (1990). Search strategies of foraging animals. Am. Sci..

[35] Stephens D.W., Krebs J.R. (2019). Foraging Theory.

[36] Crystal J.D. (2010). Episodic-like memory in animals. Behav. Brain Res..

[37] Jozet-Alves C., Bertin M., Clayton N.S. (2013). Evidence of episodic-like memory in cuttlefish. Curr. Biol..

[38] Boal J.G., Dunham A.W., Williams K.T., Hanlon R.T. (2000). Experimental evidence for spatial learning in octopuses (*Octopus bimaculoides*). J. Comp. Psychol..

[39] Hvorecny L.M., Grudowski J.L., Blakeslee C.J., Simmons T.L., Roy P.R., Brooks J.A., Hanner R.M., Beigel M.E., Karson M.A., Nichols R.H. (2007). Octopuses (*Octopus bimaculoides*) and cuttlefishes (*Sepia pharaonis*, *S. officinalis*) can conditionally discriminate. Anim. Cogn..

[40] Karson M.A., Boal J.G., Hanlon R.T. (2003). Experimental evidence for spatial learning in cuttlefish (*Sepia officinalis*). J. Comp. Psychol..

[41] Clayton N., Emery N., Dickinson A., Hurley S., Nudds M. (2006). The rationality of animal memory: Complex caching strategies of Western scrub jays. Rational Animals.

[42] Cartron L., Darmaillacq A.-S., Jozet-Alves C., Shashar N., Dickel L. (2012). Cuttlefish rely on both polarized light and landmarks for orientation. Anim. Cogn..

[43] Shaw P., Hendrickson L., McKeown N., Stonier T., Naud M.-J., Sauer W. (2010). Discrete spawning aggregations of loliginid squid do not represent genetically distinct populations. Mar. Ecol. Prog. Ser..

[44] Hall K.C., Hanlon R.T. (2002). Principal features of the mating system of a large aggregation of the giant Australian cuttlefish *Sepia apama* (Mollusca: Cephalopoda). Mar. Biol..

[45] Finn J.K., Tregenza T., Norman M.D. (2009). Defensive tool use in a coconut-carrying octopus. Curr. Biol..

[46] Montana J., Finn J.K., Norman M.D. (2015). Liquid sand burrowing and mucus utilization as novel adaptations to a structurally-simple environment in *Octopus kaurna* Stranks, 1990. Behaviour.

[47] Hartwick E., Ambrose R.F., Robinson S.M.C. (1984). Den utilization and the movement of tagged *Octopus dofleini*. Mar. Behav. Physiol..

[48] Ambrose R.F. (1982). Octopus bimaculatus. Mar. Ecol. Prog. Ser..

[49] Mather J.A. (1982). Factors affecting the spatial distribution of natural populations of *Octopus joubini* Robson. Anim. Behav..

[50] Iribarne O.O. (1990). Use of shelter by the small Patagonian octopus *Octopus tehuelchus*: Availability, selection and effects on fecundity. Mar. Ecol. Prog. Ser..

[51] Guerra Á., Hernández-Urcera J., Garci M.E., Sestelo M., Regueira M., González Á.F., Cabanellas-Reboredo M., Calvo-Manazza M., Morales-Nin B. (2014). Dwellers in dens on sandy bottoms: Ecological and behavioural traits of *Octopus vulgaris*. Sci. Mar..

[52] Anderson R.C., Hughes P.D., Mather J.A., Steele C.W. (1999). Determination of the diet of *Octopus rubescens* Berry, 1953 (Cephalopoda: Octopodidae), through examination of its beer bottle dens in Puget Sound. Malacologia.

[53] Katsanevakis S., Verriopoulos G. (2004). Den ecology of *Octopus vulgaris* Cuvier, 1797, on soft sediment: Availability and types of shelter. Sci. Mar..

[54] Leite T.S., Vidal E.A., Lima F.D., Lima S.M., Dias R.M., Giuberti G.A., de Vasconcellos D., Mather J.A., Haimovici M. (2021). A new species of pygmy *Paroctopus* Naef, 1923 (Cephalopoda: Octopodidae): The smallest southwestern Atlantic octopod, found in sea debris. Mar. Biodivers..

[55] Scheel D., Godfrey-Smith P., Lawrence M. (2016). Signal use by octopuses in an agonistic interaction. Curr. Biol..

[56] O’Brien D.A., Taylor M.L., Masonjones H.D., Boersch-Supan P.H., O’Shea O.R. (2020). Drivers of octopus abundance and density in an anchialine lake: A 30 year comparison. J. Exp. Mar. Biol. Ecol..

[57] Voss K.M., Mehta R.S. (2021). Asymmetry in the frequency and proportion of arm truncation in three sympatric California *Octopus* species. Zoology.

[58] Alupay J.S. (2013). Characterization of Arm Autotomy in the Octopus, Abdopus Aculeatus (d’Orbigny, 1834). Ph.D. Thesis.

[59] Bush S.L. (2012). Economy of arm autotomy in the mesopelagic squid *Octopoteuthis deletron*. Mar. Ecol. Prog. Ser..

[60] Staudinger M.D., Juanes F. (2010). Size-dependent susceptibility of longfin inshore squid (*Loligo pealeii*) to attack and capture by two predators. J. Exp. Mar. Biol. Ecol..

[61] Meisel D.V., Kuba M., Byrne R.A., Mather J. (2013). The effect of predatory presence on the temporal organization of activity in *Octopus vulgaris*. J. Exp. Mar. Biol. Ecol..

[62] Josef N., Amodio P., Fiorito G., Shashar N. (2012). Camouflaging in a complex environment—Octopuses use specific features of their surroundings for background matching. PLoS ONE.

[63] Hanlon R.T., Forsythe J.W., Joneschild D.E. (1999). Crypsis, conspicuousness, mimicry and polyphenism as antipredator defences of foraging octopuses on Indo-Pacific coral reefs, with a method of quantifying crypsis from video tapes. Biol. J. Linn. Soc..

[64] Mather J.A., Mather D.L. (2004). Apparent movement in a visual display: The ‘passing cloud’ of *Octopus cyanea* (Mollusca: Cephalopoda). J. Zool..

[65] Packard A., Sanders G.D. (1971). Body patterns of Octopus vulgaris and maturation of the response to disturbance. Anim. Behav..

[66] Norman M.D., Finn J., Tregenza T. (2001). Dynamic mimicry in an Indo–Malayan octopus. Proc. R. Soc. Lond. Ser. B Biol. Sci..

[67] Mäthger L.M., Bell G.R., Kuzirian A.M., Allen J.J., Hanlon R.T. (2012). How does the blue-ringed octopus (*Hapalochlaena lunulata*) flash its blue rings?. J. Exp. Biol..

[68] Caldwell R.L. (2005). An observation of inking behavior protecting adult *Octopus bocki* from predation by green turtle (*Chelonia mydas*) hatchlings. Pac. Sci..

[69] Sreeja V., Bijukumar A. (2013). Ethological studies of the veined octopus *Amphioctopus marginatus* (Taki) (Cephalopoda: Octopodidae) in captivity, Kerala, India. J. Threat. Taxa.

[70] Young R.F., Winn H.E. (2003). Activity patterns, diet, and shelter site use for two species of moray eels, *Gymnothorax moringa* and *Gymnothorax vicinus*, in Belize. Copeia.

[71] Langridge K.V. (2009). Cuttlefish use startle displays, but not against large predators. Anim. Behav..

[72] Chiao C.-C., Chubb C., Hanlon R.T. (2015). A review of visual perception mechanisms that regulate rapid adaptive camouflage in cuttlefish. J. Comp. Physiol. A.

[73] Allen J.J., Mäthger L.M., Barbosa A., Hanlon R.T. (2009). Cuttlefish use visual cues to control three-dimensional skin papillae for camouflage. J. Comp. Physiol. A.

[74] Ramirez M.D., Oakley T.H. (2015). Eye-independent, light-activated chromatophore expansion (LACE) and expression of phototransduction genes in the skin of *Octopus bimaculoides*. J. Exp. Biol..

[75] Buresch K.C., Mäthger L.M., Allen J.J., Bennice C., Smith N., Schram J., Chiao C.-C., Chubb C., Hanlon R.T. (2011). The use of background matching vs. masquerade for camouflage in cuttlefish *Sepia officinalis*. Vis. Res..

[76] Huffard C.L. (2006). Locomotion by *Abdopus aculeatus* (Cephalopoda: Octopodidae): Walking the line between primary and secondary defenses. J. Exp. Biol..

[77] Hanlon R.T., Watson A.C., Barbosa A. (2010). A “mimic octopus” in the Atlantic: Flatfish mimicry and camouflage in *Macrotritopus defilippi*. Biol. Bull..

[78] Huffard C.L., Saarman N., Hamilton H., Simison W.B. (2010). The evolution of conspicuous facultative mimicry in octopuses: An example of secondary adaptation?. Biol. J. Linn. Soc..

[79] Hanlon R.T., Conroy L.-A., Forsythe J.W. (2008). Mimicry and foraging behaviour of two tropical sand-flat octopus species off north Sulawesi, Indonesia. Biol. J. Linn. Soc..

[80] Karpestam E., Merilaita S., Forsman A. (2016). Colour polymorphism protects prey individuals and populations against predation. Sci. Rep..

[81] Zylinski S., How M., Osorio D., Hanlon R.T., Marshall N. (2011). To be seen or to hide: Visual characteristics of body patterns for camouflage and communication in the Australian giant cuttlefish *Sepia apama*. Am. Nat..

[82] Langridge K.V. (2006). Symmetrical crypsis and asymmetrical signaling in the cuttlefish *Sepia officinalis*. Proc. R. Soc. B Biol. Sci..

[83] Staudinger M.D., Hanlon R.T., Juanes F. (2011). Primary and secondary defences of squid to cruising and ambush fish predators: Variable tactics and their survival value. Anim. Behav..

[84] Mather J.A. (2010). Vigilance and antipredator responses of Caribbean reef squid. Mar. Freshw. Behav. Physiol..

[85] Mather J.A. (2016). Mating games squid play: Reproductive behaviour and sexual skin displays in Caribbean reef squid *Sepioteuthis sepioidea*. Mar. Freshw. Behav. Physiol..

[86] Mather J.A. (1986). Sand digging in *Sepia officinalis*: Assessment of a cephalopod mollusc’s “fixed” behavior pattern. J. Comp. Psychol..

[87] York C.A., Bartol I.K. (2016). Anti-predator behavior of squid throughout ontogeny. J. Exp. Mar. Biol. Ecol..

[88] Wood J.B., Pennoyer K.E., Derby C.D. (2008). Ink is a conspecific alarm cue in the Caribbean reef squid, *Sepioteuthis sepioidea*. J. Exp. Mar. Biol. Ecol..

[89] Wood J.B., Maynard A.E., Lawlor A.G., Sawyer E.K., Simmons D.M., Pennoyer K.E., Derby C.D. (2010). Caribbean reef squid, *Sepioteuthis sepioidea*, use ink as a defense against predatory French grunts, *Haemulon flavolineatum*. J. Exp. Mar. Biol. Ecol..

[90] Derby C.D., Tottempudi M., Love-Chezem T., Wolfe L.S. (2013). Ink from longfin inshore squid, *Doryteuthis pealeii*, as a chemical and visual defense against two predatory fishes, summer flounder, *Paralichthys dentatus*, and sea catfish, *Ariopsis felis*. Biol. Bull..

[91] Jiang M., Zhao C., Yan R., Li J., Song W., Peng R., Han Q., Jiang X. (2019). Continuous inking affects the biological and biochemical responses of cuttlefish *Sepia pharaonis*. Front. Physiol..

[92] Hikidi Y., Hirohashi N., Kasugai T., Sato N. (2020). An elaborate behavioural sequence reinforces the decoy effect of ink during predatory attacks on squid. J. Ethol..

[93] Anderson R.C., Mather J.A. (1996). Escape responses of *Euprymna scolopes* Berry, 1911 (Cephalopoda, Sepiolidae). J. Molluscan Stud..

[94] Helmer D., Geurten B.R., Dehnhardt G., Hanke F.D. (2017). Saccadic movement strategy in common cuttlefish (*Sepia officinalis*). Front. Physiol..

[95] Onthank K.L., Cowles D.L. (2011). Prey selection in *Octopus rubescens*: Possible roles of energy budgeting and prey nutritional composition. Mar. Biol..

[96] Ambrose R.F. (1984). Food preferences, prey availability, and the diet of *Octopus bimaculatus* Verrill. J. Exp. Mar. Biol. Ecol..

[97] Vincent T., Scheel D., Hough K. (1998). Some aspects of diet and foraging behavior of *Octopus dofleini* Wülker, 1910 in its northernmost range. Mar. Ecol..

[98] Iribarne O. (1991). Life history and distribution of the small south-western Atlantic octopus, *Octopus tehuelchus*. J. Zool..

[99] Mather J. (2011). Why is *Octopus cyanea* Gray in Hawaii specializing in crabs as prey?. Vie Milieu.

[100] Portela E., Simões N., Rosas C., Mascaró M. (2014). Can preference for crabs in juvenile Octopus maya be modified through early experience with alternative prey?. Behaviour.

[101] Schnell A.K., Boeckle M., Rivera M., Clayton N.S., Hanlon R.T. (2021). Cuttlefish exert self-control in a delay of gratification task. Proc. R. Soc. B.

[102] Kuo T.-H., Chiao C.-C. (2020). Learned valuation during forage decision-making in cuttlefish. R. Soc. Open Sci..

[103] Billard P., Clayton N.S., Jozer-Alves C. (2020). Cuttlefish retrieve whether they smelt or saw a previously encountered item. Sci. Rep..

[104] Billard P., Schnell A.K., Clayton N.S., Jozet-Alves C. (2020). Cuttlefish show flexible and future-dependent foraging cognition. Biol. Lett..

[105] Anderson R.C., Wood J.B., Mather J.A. (2008). *Octopus vulgaris* in the Caribbean is a specializing generalist. Mar. Ecol. Prog. Series.

[106] Scheel D., Leite T., Mather J., Langford K. (2017). Diversity in the diet of the predator *Octopus cyanea* in the coral reef system of Moorea, French Polynesia. J. Nat. Hist..

[107] Mather J.A., Leite T.S., Batista A.T. (2012). Individual prey choices of octopuses: Are they generalist or specialist?. Curr. Zool..

[108] Scheel D., Anderson R. (2012). Variability in the diet specialization of *Enteroctopus dofleini* (Cephalopoda: Octopodidae) in the eastern Pacific examined from midden contents. Am. Malacol. Bull..

[109] Ambrose R.F., Nelson B.V. (1983). Predation by *Octopus vulgaris* in the Mediterranean. Mar. Ecol..

[110] Nixon M., Maconnachie E. (1988). Drilling by *Octopus vulgaris* (Mollusca: Cephalopoda) in the Mediterranean. J. Zool..

[111] Wodinsky J. (1969). Penetration of the shell and feeding on gastropods by *Octopus*. Am. Zool..

[112] Wodinsky J. (1973). Mechanism of hole boring in *Octopus vulgaris*. J. Gen. Psychol..

[113] Steer M.A., Semmens J.M. (2003). Pulling or drilling, does size or species matter? An experimental study of prey handling in *Octopus dierythraeus*. J. Exp. Mar. Biol. Ecol..

[114] Fiorito G., Gherardi F. (1999). Prey-handling behaviour of *Octopus vulgaris* (Mollusca, Cephalopoda) on bivalve preys. Behav. Process..

[115] Blustein D.H., Anderson R.C. (2016). Localization of octopus drill holes on cowries. Am. Malacol. Bull..

[116] Anderson R.C., Mather J.A. (2007). The packaging problem: Bivalve prey selection and prey entry techniques of the octopus *Enteroctopus dofleini*. J. Comp. Psychol..

[117] Merlino B. (2013). Predation Behavior of *Octopus joubini* Cuvier. Master’s Thesis.

[118] Dickel L., Chichery M.-P., Chichery R. (1998). Time differences in the emergence of short- and long-term memory during post-embryonic development in the cuttlefish, Sepia. Behav. Process..

[119] Wells M., Wells J. (1958). The effect of vertical lobe removal on the performance of octopuses in retention tests. J. Exp. Biol..

[120] Muntz W., Sutherland N., Young J. (1962). Simultaneous shape discrimination in octopus after removal of the vertical lobe. J. Exp. Biol..

[121] Burghardt G.M., Hess E.H. (1966). Food imprinting in the snapping turtle, *Chelydra serpentina*. Science.

[122] Darmaillacq A.-S., Jozet-Alves C., Bellanger C., Dickel L., Darmaillacq A.-S., Dickel L., Mather J. (2014). Cuttlefish preschool or how to learn in the peri-hatching period. Cephalopod Cognition.

[123] Darmaillacq A.-S., Chichery R., Dickel L. (2006). Food imprinting, new evidence from the cuttlefish *Sepia officinalis*. Biol. Lett..

[124] Poirier R., Chichery R., Dickel L. (2004). Effects of rearing conditions on sand digging efficiency in juvenile cuttlefish. Behav. Process..

[125] Romagny S., Darmaillacq A.-S., Guibé M., Bellanger C., Dickel L. (2012). Feel, smell and see in an egg: Emergence of perception and learning in an immature invertebrate, the cuttlefish embryo. J. Exp. Biol..

[126] Darmaillacq A.-S., Lesimple C., Dickel L. (2008). Embryonic visual learning in the cuttlefish, *Sepia officinalis*. Anim. Behav..

[127] Lee Y.-H., Yan H.Y., Chiao C.-C. (2012). Effects of early visual experience on the background preference in juvenile cuttlefish *Sepia pharaonis*. Biol. Lett..

[128] Robin J.-P., Roberts M., Zeidberg L., Bloor I., Rodriguez A., Briceno F., Downey N., Mascaro M., Navarro M., Guerra A. (2014). Transitions during cephalopod life history: The role of habitat, environment, functional morphology and behaviour. Adv. Mar. Biol..

[129] Wilson R.C., Bonawitz E., Costa V.D., Ebitz R.B. (2021). Balancing exploration and exploitation with information and randomization. Curr. Opin. Behav. Sci..

[130] Degen J., Hovestadt T., Storms M., Menzel R. (2018). Exploratory behavior of re-orienting foragers differs from other flight patterns of honeybees. PLoS ONE.

[131] Tchernichovski O., Benjamini Y. (1998). The dynamics of long term exploration in the rat: Part II. An analytical model of the kinematic structure of rat exploratory behavior. Biol. Cybern..

[132] Burghardt G.M. (2005). The Genesis of Animal Play: Testing the Limits.

[133] O’Hara M., Auersperg A.M. (2017). Object play in parrots and corvids. Curr. Opin. Behav. Sci..

[134] Mather J.A., Anderson R.C. (1999). Exploration, play and habituation in octopuses (*Octopus dofleini*). J. Comp. Psychol..

[135] Kuba M.J., Byrne R.A., Meisel D.V., Mather J.A. (2006). When do octopuses play? Effects of repeated testing, object type, age, and food deprivation on object play in *Octopus vulgaris*. J. Comp. Psychol..

[136] Pisula W. (2008). Play and exploration in animals—A comparative analysis. Pol. Psychol. Bull..

[137] Peters A., McEwen B.S., Friston K. (2017). Uncertainty and stress: Why it causes diseases and how it is mastered. Prog. Neurobiol..

